# A review of efficacy and safety of Ugandan anti-malarial plants with application of RITAM score

**DOI:** 10.1186/s12936-023-04486-6

**Published:** 2023-03-17

**Authors:** Jimmy R. Angupale, Jonans Tusiimire, Ndidi C. Ngwuluka

**Affiliations:** 1 Department of Pharmaceutical Sciences, Faculty of Medicine, Mbarara University of Science and Technology, P.O Box 1410, Mbarara, Uganda; 2 Pharm-Biotechnology and Traditional Medicine Centre, Mbarara University of Science and Technology, P.O Box 1410, Mbarara, Uganda; 3 Department of Pharmacy, Faculty of Medicine, Mbarara University of Science and Technology, P.O Box 1410, Mbarara, Uganda; 4 Department of Pharmaceutics, Faculty of Pharmaceutical Sciences, University of Jos, Jos, Nigeria

**Keywords:** RITAM Score, Uganda, Antimalarial plants, Efficacy, Safety

## Abstract

**Background:**

Malaria, a treatable disease mainly caused by *Plasmodium falciparum* has remained a health challenge in Africa, a continent that accounted for 96% of total global cases and deaths in 2021. Uganda, a malaria endemic country is experiencing malaria parasite resistance to some of the drugs used in the artemisinin-based combination therapy (ACT). In an effort to prioritize herbal medicines for new product development, this review synthesized the available safety and efficacy literature on the Ugandan anti-malarial plants to suggest most effective herbal plants.

**Methods:**

Literature was exhaustively searched using engines and databases, such as Google scholar, Pubmed, and Scopus-indexed journals during the period of June 2020–December 2021. In the first phase, information on ethnobotanical uses of anti-malarial plants in Uganda was gathered and synthetized to generate a list of plants, followed by data on anti-malarial efficacy (both in vitro and in vivo) on each listed plant. Minimum inhibitory concentrations (µg/ml), and % parasite suppression for every plant were scored using The Research Initiative on Traditional and Antimalarial Methods (RITAM) scoring system. The best twenty (20) plants were evaluated for acute safety (LD_50_) data in rat model, plant parts used, ease of cultivation, presence of clinical studies and other relevant factors for suggesting the best three (3) plants for future anti-malarial product development.

**Results:**

Over one hundred twenty-six (126) plant species are used in Uganda for treatment of malaria in local communities. Out of these, about 33% (41) have been studied for efficacy and safety, with *Artemisia annua* and *Vernonia amygdalina* being the most extensively studied and among the best twenty (20) anti-malarial plants in Uganda. Both are limited by parasite recrudescence in clinical studies. *Microglossa pyrifolia*, a very potent plant (IC50 = 0.03 – 0.05 µg/ml has potential to penetrate the liver and could ameliorate the challenge of recrudescence if combined with *A. annua* and *V. amygdalina* in a polyherbal formulation.

**Conclusion:**

There are many plants with promising potential for malaria treatment in Uganda and a herbal combination of *A. annua*, *V. amydalina* and *M. pyrifolia* could offer the next herbal ACT if carefully studied and developed.

## Background

Malaria is a treatable disease but life threatening, causing acute febrile illness [1]. The endemic has continued to cripple the global population with children under five years and the African continent carrying the highest disease burden over the years. The World Health Organization (WHO) reported increasing malaria cases from 2019 to 2021 (227, 241, and 247 million cases in 2019, 2020 and 2021, respectively) in eighty-five (85) endemic countries [2–4]. The WHO African region accounted for 94% of the cases in 2019, 95% in both 2020 and 2021, and four African countries (Nigeria—31.3%, Democratic Republic of Congo—12.6%, United Republic of Tanzania—4.1%, and Niger—3.9%) were responsible for half of the total global cases in 2021 [2]. Even though the rise in malaria infections has been attributed to disruptions in the health care system in 2019 by Covid-19 pandemic (by 6% in 2020) [5], the African region has remained off-track from Global Technical Strategy (GTS) 2020 targets for reduction of malaria cases by 45%.

Uganda is among the six (6) African countries that accounted for 55% of the global malaria cases, a disease now termed essentially as an African problem since the continent accounts for over 95% of total global cases and 96% of all deaths [6] According to the Uganda Ministry of Health malaria report 2017–2018, Uganda remains one of the ten (10) countries in sub-Saharan Africa, accounting for 70% of the global malaria cases and deaths [7]. The country also ranked 5th in terms of global malaria morbidity and 9th for malaria mortality in 2018 according to World Health Organization [8]. Uganda also accounted for 5% and 5.1% of the global malaria cases in 2020 and 2021 respectively [5]. In addition, a malaria incidence rate of 42.4% was also reported in 2020 in Apac district (Uganda) from children between the ages of 1 to 4 years [9]. Based on these statistics, malaria is still a serious health challenge in Uganda and other African countries, and requires more efforts to realize GTS target of reducing its global incidence and mortality rates by at least 90% by 2030.

 Artemisinin-based combination therapy (ACT) remains the gold standard of malaria treatment in Uganda. ACT was adopted as first-line treatment for malaria in Uganda in 2004 and this policy change has proven to be very vital in the reduction of malaria death tolls in the country [10, 11]. Currently, reports of resistance of the malaria parasite *Plasmodium falciparum* against Artemisinin-based combinations have surfaced in East Africa, mainly Uganda and Rwanda [12]. There was a decreased susceptibility to artemether–lumefantrine in Northern and Eastern Uganda associated with multiple polymorphisms, notably *Pfkelch13* 469Y mutations against lumefantrine [12–14]. Though these mutations are associated with delayed parasite clearance by ACT, the researchers did not find any association between *Pfkelch13* nonsynonymous mutations and delayed parasite clearance, with exception of one patient. In addition to the mutation against artemisinin, deletions in *pfhrp2* and *pfhrp3* (*pfhrp2/3*) genes that render the parasite undetectable by RDT have also been reported [3]. Another R622I mutation occurred independently in Africa, having been found in Eritrea, Ethiopia, Somalia and Sudan, and with increasing frequency in the Horn of Africa (Somali Peninsula). These findings are reaffirming the continued evolution in the phenotypes and genotypes of the *Plasmodium* parasite in Africa, thus raising alarm of increase such cases of resistance in near future. Therefore, there is the need for more research into the development of new therapeutics and other innovative approaches as alternatives to combat this African health problem.

In Uganda, the use of herbal medicines among local communities (60–80%) for management of malaria and other disease conditions continues to offer reliable alternatives for new product development [15]. These ethnobotanical uses have also been supported by several preclinical (in vitro and in vivo animal studies) and clinical efficacy studies. Safety data on most of these plants have also been generated over the years. With the complexity of phytochemical compounds in a single crude extract, plant-based therapies have minimal susceptibility to microbial resistance always associated with synthetic drugs, including ACT, which are based on individual chemical compounds. In addition, positive synergistic and/or clinically beneficial interactions among different chemicals in one extract have also been reported [16]. An example of such is increased absorption of artemisinin by other components of *Artemisinia annua* tea [17]. Therefore, selection of the most efficacious anti-malarial plant phyto-extracts for dosage form standardization using latest and advanced formulation principles/technologies is key for addressing the treatment challenges presented by this endemic disease.

Previous reviews on anti-malarial plants in Uganda [18], mainly focused on ethnobotanical surveys which usually capture frequency of use and a summary of existing literature on the phyto-compounds and efficacy, without objective analysis of these information to select the most efficacious. The Research Initiative on Traditional and Antimalarial Methods (RITAM) founded in 1999 designed a standard score criteria that is useful for analysing literature on anti-malarial plants [19]. Based on the RITAM score system, each plant (or herbal remedy) is given a numerical value (score) based on frequency of ethnobotanical citations, laboratory efficacy in vitro, in vivo, and safety. This method is objective and can rigorously guide in selection of the most efficacious but safe plants for further anti-malarial product development.

Therefore, this review synthesized the available safety and efficacy literature on the Ugandan anti-malarial plants to reliably suggest the highly ranking ones for subsequent herbal product innovations based on scientifically validated criteria.

## Methods

A comprehensive literature search was conducted using available databases including Pubmed, Google scholar, and Scopus-indexed journals. Objective keywords such as ethnobotanical surveys, anti-malarial plants in Uganda, and others were used to get peer-reviewed articles on the herbal remedies mentioned for malaria treatment. Only ethnobotanical studies on anti-malarial plants in Uganda were included in the review. For each survey included in the study, at least 5–10 plants with the highest frequency of use were considered to generate a list of potential anti-malarial plants.

In the second phase, further searches were exhaustively done on each listed plant with specificity on plant name, anti-plasmodial activity, in vitro anti-malarial efficacy, in vivo anti-malarial efficacy, safety profile, LD_50_, and acute toxicity. Priority was given to the studies conducted on plants collected from Uganda that have met the minimal quality requirements of data rigour, methods and scientific validity. Only safety studies that reported the minimum lethal dose (LD_50_ in mg/kg) in animals (rat model) were included. RITAM score system was adapted with modifications [19]. The scores were awarded to each plant employed in studies that have demonstrated the best efficacy from in vitro to in vivo with considerations of the solvent and the parts of the plant used for extraction, as summarized in Table 1. Plants with no scientific data on efficacy were excluded at this stage. The total score for each plant were calculated and they were ranked based on values from the highest to the lowest.

**Table 1:** Article inclusion and exclusion criteria for RITAM efficacy scoring

Parameters	Inclusion criteria	Exclusion criteria
Part of the plant studied (Leaves, Stembark, Roots, Fruits, Flowers)	Leaves, Fruits, Stembark, Seeds (In case of activities reported on different parts, the one with best activity is considered for scores)	Flowers and Roots
Extraction solvent—Aqueous., Petroleum Ether, Dichloromethane, Ethanol, Methanol)	The one which gives the best activity	The one with less activity
Location of the study plant part collection (Uganda, E. Africa, Africa)	In Uganda and from all other parts if the active compound is known	Outside Uganda and active anti-malarial compound is not known for the plant species
RITAM SCORE [19]		
Test	Test Result Range	Score
In vitro Antiplasmodial Test (µg/mL)	Not tested	0
	< 2	10
	2.0 – 5.0	5
	5.1 – 10	3
	11 – 25	2
	26 – 50	1
	Activity confirmed in more than one strain of P. falciparum	2
In vivo Antiplasmodial Test in mice (% inhibition)	Not tested	0
	100 – 90	10
	90 – 50	9 – 5
	50 – 10	5 – 1
	0	-2

In the third phase, the twenty best plants selected from phase two were further assessed for information on the plant parts used, extraction solvent system, ease of plant cultivation, safety level, confirmation of preclinical efficacy in clinical trials and exclusiveness of the potency reported. These assessments were interpreted and summarized for the selection of the best three plants as crude actives for possible development of efficacious, cost effective and commercially sustainable anti-malarial products.

## Results and discussion

### Ugandan anti-malarial plants

Various ethnobotanical survey studies have been conducted in Uganda on the plants locally used for malaria. Extensive review of ethnobotanical surveys in the country with special consideration for the different geographical regions (Eastern, Western, Central and Northern) [18] revealed fifteen plant species as the most commonly used in Uganda. These plants included *Bidens pilosa*, *Tithonia diversifolia*, *Vernonia amygdalina*, *Vernonia lasiopus*, *Carica papaya*, *Hoslundia opposita*, *Mangifera indica*, *Cymbopogon citratus*, *Justicia betonica*, *Markhamia lutea*, *Moringa oleifera*, *Aristolochia elegans*, *Cajanus cajan*, *Toddalia asiatica*, and *Azadirachta indica*.

An ethnobotanical survey conducted on the plants used for treatment of malaria in Mpigi district documented eighty-six plant species [20]. Among these, the most commonly reported with Fr (Frequency of report) values from 38 to 17 included (in descending order) *V. amygdalina*, *B. pilosa*, *J. betonica*, *Microglossa pyrifolia*, *Clerodendrum rotundifolium*, *V. lasiopus*, *Aloe dawei*, *Leonotis nepetofolia*.

In yet another similar study conducted in Butebo County, Eastern Uganda [21], thirty-three plant species were documented, but the eight most common ones with PPK (percentage of people who have knowledge about the use of a species in the treatment of malaria) values ranging from 90 to 70% in the descending order include *Chamaecrista nigricans*, *Zanthoxylum chalybeum*, *Schkuhria pinnata*, *Ocimum basilicum*, *Euclea latideus*, *Erythrina abyssinica*, *A. indica*, and *Ocoba spinosa*.

Another ethnobotanical survey was also carried out in Budondo sub-county located in Jinja district north-east of Kampala [22]. From the study, a total of thirty-seven plant species were documented for treatment of malaria. Among these, the most common with percentage of mention ranging from 64.8 to 15.4% included *V. amygdalina*, *Aloe vera*, *Callistermon citrinu*s, *Mormodica foetida*, *Cyphostemma adenocaule*, and *Eucalyptus globulus*.

In Kamuli district, Eastern Uganda [23], twenty-seven plant species for treatment of malaria were reported. *V. amygdalina*, *M. foetida*, *Z. chalybeum*, *Lantana camara*, *M. indica*, and *Chenopodium ambrosioïdes*, were the most frequently mentioned species. Another ethnobotanical survey was also conducted in Mbarara district, western Uganda [24]. From this study, a total of twenty plant species were documented and eight considered to be the most commonly used with frequency of mention ranging from 102 to 24 included (in descending order): *V. amygdalina*, *Pseudarthria hookeri*, *C. rotundifolium*, *Lantana trifolia*, *T. asiatica*, *V. lasiopus*, and *Erlangea cordifolia*.

In Cegere sub-county, Apac district, northern Uganda, a total of 20 plant species were documented for preventing and treating malaria in the area [25], and seven most commonly used with citation frequency ranging from 69 to 7 (in descending order) include *S. pinnata*, *Baccharoides adoensis*, *A. indica*, *Crotalaria ochroleuca*, *A. vera*, *M. oleifera*, and *Curcuma longa*.

Anti-malarial plants used in the areas of Abukamola, Angeta, Oculokori, and Omarari of Alebtong district (Northern Uganda) were also documented [26]. A total of forty-three plant species were reported and the most common with PRK values ranging from 23.5 to 9.9% included *Clerodendrum umbellatum*, *Canthium lactescens*, *Crotolaria laburnifolia*, *Chasmanthera dependens*, *Chamaecrista hildbrandtii*, and *Securidaca longipenduculata*.

Another study which captured ethnomedicinal use, preference for species and ecological viability of plants used for treatment of malaria was earlier conducted among the communities living around the Sango Bay Forest Reserve in southern Uganda [27]. Sixteen plant species were unveiled and the five most common ranked basing on the importance index (which focuses on the level of relevance attached to each plant by the respondents for management of the disease under investigation) included *Hallea rubrostipulata*, *V. amygdalina*, *Warburgia ugandensis*, *Syzygium guineense*, and *Z. chalybeum.*

Tugume et al. [28] also documented all the medicinal plants in Mabira Central Forest Reserve (CFR) in Central Uganda. According to the researchers, the thirteen most important medicinal plants for treatment of malaria included *V. amygdalina*, *M. feotida*, *Indigofera congesta*, *Solanum nigrum*, *A. vera*, *Hoslundia opposita*, *Markhamia lutea*, *V. lasiopus*, *Melanthera scandens*, *Aristolochia elegans*, *Alstonia boonei*, and *J. betonica.*

Furthermore, a total of fifty-six plant species were reported to be used for treatment of malaria in Nyakayojo sub-county in south western Uganda [29]. Among them, the fourteen most commonly reported included *V. amygdalina*, *Aloe* sp., wild sp., *J. betonica*, *Vernonia adoensis*, *T. diversifolia*, *A. indica*, *Clutia abyssinica*, *V. lasiopus*, *Solanecio mannii*, *M. pyrifolia*, *Bothriocline longipes*, *Conyza bonariensis*, *Guizotia scabra*, and *Gynura scandens*.

In a comprehensive literature review on the plants used for treatment of malaria in Uganda, approximately 182 plant species were documented. Among these, 112 plant species, including *Artemisia annua,* were reported to have been investigated for anti-malarial activities, with 96% showing positive results. These tested plants were compared and sorted with the plants reported above in the various ethnobotanical surveys from different parts of the country to generate a list of about 126 plant species [30]. This implies that there are over 126 plant species currently used by local communities in Uganda for management of malaria and the majority of them have been tested.

### Efficacy of selected anti-malarial studies

The standard RITAM criterion for selection of the most efficacious and safe anti-malarial plants which consists of numerical values allocated for in vitro anti-plasmodial IC_50_ (mg/ml), percentage chemosuppression (%) in mice model and acute toxicity (LD_50_) in the rat model was adapted. Out of the 126 Ugandan anti-malarial plants, only 41 plant species were selected for the RITAM score and their rankings are as indicated in Table 2. Four (4) different rankings were created based on: (1) total score of efficacy (both MIC and % parasite suppression) and safety (LD_50_); (2) total efficacy score only; (3) in vitro score (MIC) only; and (4) in vivo score only. The second ranking is the most suitable for precise rating of the plants based exclusively on efficacy. A summary of the subsequent analysis of the best 20 plants selected is presented in Table 3.

**Table 2:** RITAM scoring analysis and ranking of selected Ugandan anti-malarial plants based on efficacy and safety data

Plant Position	Efficacy & Safety (Rank 1)		Efficacy Only (Rank 2, in vitro + in vivo)		In vitro only (Rank 3, MIC)		In vivo only (Rank 4, % suppression)	
	Plant	Score	Plants	Score	Plants	Score	Plants	Score
1	*Artemisia annua* [39, 44, 56]	29	*Artemisia annua*	23	*Artemisia annua*	13	*Artemisia annua*	10
2	*Azadirachta indica* [79–81]	26	*Momordica foetida*	22	*Azadirachta indica*	13	*Alchornea cordifolia*	10
3	*Curcuma longa* [82, 83]	25	*Azadirachta indica*	20	*Curcuma longa*	13	*Bidens pilosa*	10
4	*Carica papaya* [84–86]	24	*Toddalia asiatica*	20	*Carica papaya*	13	*Moringa oleifera*	9
5	*Moringa oleifera* [87–89]	23	*Curcuma longa*	19	*Momordica foetida*	13	*Momordica foetida*	9
6	*Alchornea cordifolia* [90 , 91]	21	*Tithonia diversifolia*	19	*Tithonia diversifolia*	13	*Cymbopogon citratus*	8
7	*Momordica foetida* [72, 92]	21	*Carica papaya*	18	*Microglossa pyrifolia*	13	*Toddalia asiatica*	8
8	*Artemisia afra* [93, 94]	20	*Moringa oleifera*	17	*Toddalia asiatica*	12	*Aspilia africana*	8
9	*Bidens pilosa* [95–97]	20	*Alchornea cordifolia*	15	*Hoslundia opposite*	12	*Ageratum conyzoides*	8
10	*Cymbopogon citratus* [98–100]	20	*Artemisia afra*	14	*Clerodendrum rotundifolium*	12	*Vernonia amygdalina*	7
11	*Toddalia asiatica* [101]	20	*Bidens pilosa*	14	*Acacia nilotica*	10	*Azadirachta indica*	7
12	*Aspilia africana* [102, 103]	19	*Cymbopogon citratus*	14	*Albizia zygia*	10	*Artemisia afra*	7
13	*Tithonia diversifolia* [66, 76, 104]	19	*Aspilia africana*	14	*Hallea rubrostipulata*	10	*Ajuga remota*	7
14	*Warburgia ugandensis*, [105, 106]	19	*Vernonia amygdalina*	13	*Albizia coriaria*	10	*Curcuma longa*	6
15	*Vernonia amygdalina* [61, 64, 66]	18	*Warburgia ugandensis*	13	*Moringa oleifera*	8	*Tithonia diversifolia*	6
16	*Ajuga remota* [107]	18	*Ageratum conyzoides*	13	*Artemisia afra*	7	*Warburgia ugandensis*	6
17	*Hoslundia opposita* [108, 109]	18	*Microglossa pyrifolia*	13	*Warburgia ugandensis*	7	*Solanum nigrum*	6
18	*Solanum nigrum* [110, 111]	17	*Ajuga remota*	12	*Harrisonia abyssinica*	7	*Carica papaya*	5
19	*Acacia nilotica* [112]	16	*Hoslundia opposita*	12	*Cymbopogon citratus*	6	*Ocimum basilicum*	5
20	*Ageratum conyzoides* [113, 114]	16	*Clerodendrum rotundifolium*	12	*Aspilia africana*	6	*Zanthoxylum chalybeum*	4
21	*Albizia zygia* [110, 115, 116]	16	*Solanum nigrum*	11	*Vernonia amygdalina*	6	*Maytenus senegalenses*	4
22	*Hallea rubrostipulata* [117, 118]	16	*Acacia nilotica*	10	*Zanthoxylum chalybeum*	6	*Erythrina abyssinica*	3
23	*Ocimum basilicum* [99, 119]	14	*Albizia zygia*	10	*Erythrina abyssinica*	6	*Vernonia lasiopus*	1
24	*Microglossa pyrifolia* [72]	13	*Hallea rubrostipulata*	10	*Alchornea cordifolia*	5	*Hoslundia opposita*	0
25	*Zanthoxylum chalybeum* [120–122]	13	*Zanthoxylum chalybeum*	10	*Ajuga remota*	5	*Acacia nilotica*	0
26	*Clerodendrum rotundifolium* [72]	12	*Albizia coriaria*	10	*Solanum nigrum*	5	*Albizia zygia*	0
27	*Cajanus cajan* [123]	11	*Erythrina abyssinica*	9	*Ageratum conyzoides*	5	*Hallea rubrostipulata*	0
28	*Teclea nobilis* [124, 125]	11	*Ocimum basilicum*	8	*Cajanus cajan*	5	*Microglossa pyrifolia*	0
29	*Erythrina abyssinica* [126]	9	*Maytenus senegalenses*	8	*Teclea nobilis*	5	*Clerodendrum rotundifolium*	0
30	*Vernonia lasiopus* [122, 123, 127–129]	9	*Harrisonia abyssinica*	7	*Baccharoides adoensis*	5	*Cajanus cajan*	0
31	*Maytenus senegalenses* [130–132]	8	*Cajanus cajan*	5	*Tagetes minuta*	5	*Teclea nobilis*	0
32	*Antiaris toxicaria* [124, 133]	7	*Teclea nobilis*	5	*Bidens pilosa*	4	*Antiaris toxicaria*	0
33	*Harrisonia abyssinica* [134]	7	*Baccharoides adoensis*	5	*Maytenus senegalenses*	4	*Harrisonia abyssinica*	0
34	*Leonotis nepetifolia* [124, 135]	7	*Tagetes minuta*	5	*Ocimum basilicum*	3	*Leonotis nepetifolia*	0
35	*Baccharoides adoensis* [136]	5	*Vernonia lasiopus*	4	*Vernonia lasiopus*	3	*Baccharoides adoensis*	0
36	*Tagetes minuta* [137]	5	*Aloe dawei*	3	*Aloe dawei*	3	*Tagetes minuta*	0
37	*Albizia coriaria* [138]	3	*Markhamia lutea*	3	*Markhamia lutea*	3	*Albizia coriaria*	0
38	*Aloe dawei* [139]	3	*Justicia betonica*	2	*Justicia betonica*	2	*Aloe dawei*	0
39	*Markhamia lutea* [124]	3	*Lantana trifolia*	2	*Lantana trifolia*	2	*Markhamia lutea*	0
40	*Justicia betonica* [140]	2	*Antiaris toxicaria*	1	*Antiaris toxicaria*	1	*Justicia betonica*	0
41	*Lantana trifolia* [127]	2	*Leonotis nepetifolia*	1	*Leonotis nepetifolia*	1	*Lantana trifolia*	0

**Table 3:** Evaluation of the best 20 selected plants from the RITAM Score of efficacy (Rank 2 from Table 2)

S/N	Plant	RITAM Score	Plant part	Extraction Solvent	Easy of cultivation	Active Principle	LD50(mg/kg)	Evidence of Clinical Trials	In vivo Dose	Other Relevant Comments
1	*Artemisia annua*	23	Leaf	Aq. + PE	easy	Artemisinin	> 5000	Yes	200	The active is widely recognized and accepted for marketing as a drug
2	*Momordica foetida*	22	Leaf	Aq	Common and easy to cultivate (climber)	Phenolic glycosides isolated but not widely studied	None	No	500	In vivo dose is high and no reports on LD_50_ and the extract mentioned to be hepatotoxic toxicity
3	*Azadirachta indica*	20	Leaf	Aq. & MeOH	Tree but leaves are easy to get	Azadirachtin	> 2000	No	250	Scored poorly in a Ugandan in vivo study
4	*Toddalia asiatica*	20	Fruit	EtoAc	Easy to cultivate in warmer areas	Nitidine	> 1000	No	500	Fruits are seasonal and not easy for purposes of commercialization
5	*Curcuma longa*	19	Rhizome	Et	Readily available	curcumin	> 5000	Yes- on bioavailability	50	Obstacles of bioavailability have limited its marketing as a drug
6	*Tithonia diversifolia*	19	Leaf	CH2CL2/Et	Readily available and easy to cultivate	Tagitinin C	> 1600	None	250	Good efficacy, even in aqueous solvents and activity confirmed in vivo in Uganda
7	*Carica papaya*	18	Leaf	Hexane & Et	Already under cultivation	Carpain, Linoleic acid	> 2000	None	400	In vivo study was conducted using ethanolic extract
8	*Moringa oleifera*	17	Leaf	MeOH/Et	Tree but leaves are easy to get	quercetin & Kaemferol	6000 mg/kg	None	200	Good efficacy and safety, and this has confirmed in Uganda
9	*Alchornea cordifolia*	13	Leaf	Aq. & MeOH	A srub easy to cultivate. Already under cultivation in Congo	Ellagic acid	5000	None	240	
10	*Vernonia amygdalina*	15	Leaf	Aq	widely grown and available	vernolide & vernodalol	2000	Yes with 67% effectiveness on day 14 (Adequate Clinical outcome)	400	Already exhibited good results clinically and studies in Uganda have proved its efficacy in rats and the extraction is cheaper
11	*Artemisia afra*	14	Leaf	CH_2_Cl_2_& MeOH	Easy to cultivate	Two guaianolide sesquiterpene lactones	> 2500	Yes but article retracted	100	May be close to Artemisia and studies reported use only non-polar solvents
12	*Bidens pilosa*	14	Leaf	CH3Cl/EtoAc	Easy	Acetylene & Flavonoids isolated but not tested	> 4000	None	500	No promising efficacy evidence in vivo
13	*Cymbopogon citratus*	14	Leaf	EtoAc/Et	Easy	Citral, myrcene and citronellal	> 2000	None	400	Scored poorly in a Ugandan In vivo study
14	*Aspilia africana*	14	Leaf	EtoAc/Et/Aq	Common weed readily available on a cultivated land	None	> 6000	Yes- reported effectivess of 70%	300	Clinical evidence available but no information on the active principle
15	*Warburgia ugandensis*	13	Stem bark	CH_3_Cl	Tree which has being cultivated	None	> 5000	No	200	The Stembark is hard to get in large quantities for this plant and the studies included are from Kenya
16	*Ageratum conyzoides*	13	Aerial/leaf	CH_2_Cl_2_ & Aq	Available herb	Isolated compounds not effective	> 5000	No	400	No studies in Uganda and the active principle not clearly known
17	*Microglossa pyrifolia*	13	Leaf	Aq	Readily available herb	Friedalanol	No information	No	No in vivo study	The plant is reported to have extremely potent activity in vitro in Uganda and a similar activity confirmed in many other studies outside Uganda (esp. Kenya) but no data in vivo and LD50 not done though there is data reports on hepatotoxicity
18	*Ajuga remota*	12	Aerial/leaf	CH_2_Cl_2_	Not common in Uganda	ajugarin-1	> 2000	No	100	Non polar solvent (dichloromethane) used for its extraction
19	*Hoslundia opposita*	12	leaf	hydro-Et/MeOH	Readily available in Uganda	hosludin, hosludal and hosludiol	> 5000	No	No in vivo study	No in vivo studies and moderate anti-plasmodial activity in other strains
20	*Clerodendrum rotundifolium*	12	leaf	Aq	Not common	no information	No information	No	No in vivo study	Has excellent in vitro potency but no in vivo studies to back it, and it is also not commonly available in case of commercialization

*Aq.* Aqueous, *PE* Petroleum ether, *MeOH* Methanol, *EtoAc* Ethyl acetate, *Et* Ethanol

### Selection of plants for possible polyherbal anti-malarial therapy

Based exclusively on the RITAM score and considering the available data on efficacy (both in vitro and in vivo), the twenty plants (Table 3) form the list of the best 20 potential anti-malarial plants in Uganda which could be enrolled for further studies in antimalarial herbal product developments. Further review of the literature on these plants revealed that only five (5) plants including *A. annua*, *V. amygdalina*, *C. longa*, *Artemisia afra*, and *Aspilia africana* have been studied up to clinical level. Among these, *A. annua* and *V. amygdalina* are the most extensively studied and their active compounds are known, isolated, and some already synthetized [31, 32]. In a relatively similar analysis which adhered to all the aspects of RITAM score including clinical correlations, *A. annua*, and *V. amygdalina* ranked as the best two anti-malarial plants [19]. The available clinical study on *C. longa* focused on pharmacokinetics (mainly bioavailability) and it showed that, there was limited bioavailability of the plant’s active anti-malarial compound curcumin [33, 34]. The clinical study of *A. afra* was later retracted due to data irregularities [35]. *Aspilia africana* demonstrated 70% effectiveness in malaria treatment [36], but there is no information on the active compounds responsible for this efficacy to guide product formulation standardization (no chemical markers known). Though extensively studied, the clinical efficacy of *A. annua* and *V. amydalina* are both limited by parasite recrudescence which hampers complete remission of the parasite from the body [37, 38]. Studies have also been done on the combination of two plants, but parasite recrudescence persisted [39]. Therefore, there is a need to conduct more clinical studies on the other plants in the best 20 list (Table 3), or search for other combinations that can eliminate the parasite recrudescence, which is associated with the inability of actives to penetrate the liver (the organ harbouring the parasites) after blood parasite clearance and reduced drug concentrations in blood [40, 41].

Interestingly, additional literature searches on the other plants in the best 20 list (Table 3), indicated that *M. pyrifolia* (aqueous extract) is one of the plants with most potent anti-plasmodial activity. It obtained maximum RITAM score for in vitro findings (Table 2, Rank 3). It also ranked third best among known anti-malarial plants in the Africa based on in vitro anti-plasmodial potency in a systemic review [42]. The plant also causes liver toxicity, indicative of the ability of the actives to penetrate the liver [43]. It is, therefore, possible that addition of this plant in lower doses to a combination of *A. annua*, and *V. amygdalina* could address the challenge of parasite recrudescence without causing liver toxicity since polyherbalism has advantages of minimizing toxic effects of certain active ingredients [16].

As much as RITAM score is important for prioritizing plants for further anti-malarial studies, it cannot be used alone since it does not adequately capture other factors, such as plant parts used, extraction solvent (aqueous preferred), and ease of cultivation. These are important in natural product development to foresee the cost of production and the possibility of commercialization. Therefore, based on current literature of the efficacy (at in vitro, mice and clinical levels), safety profile and other factors indicated in Table 3, *A. annua*, *V. amygdalina* and *M. pyrifolia* could offer a promising alternative of natural and herbal combination therapy, but needs anti-malarial activity optimization study. However, this selection does not rule out the relevance of the other plants in our list of 20 as equally potential plants for further anti-malarial studies.

### Description of the selected plants for development of herbal drug delivery systems

#### Artemisia annua

**Fig. 1:**
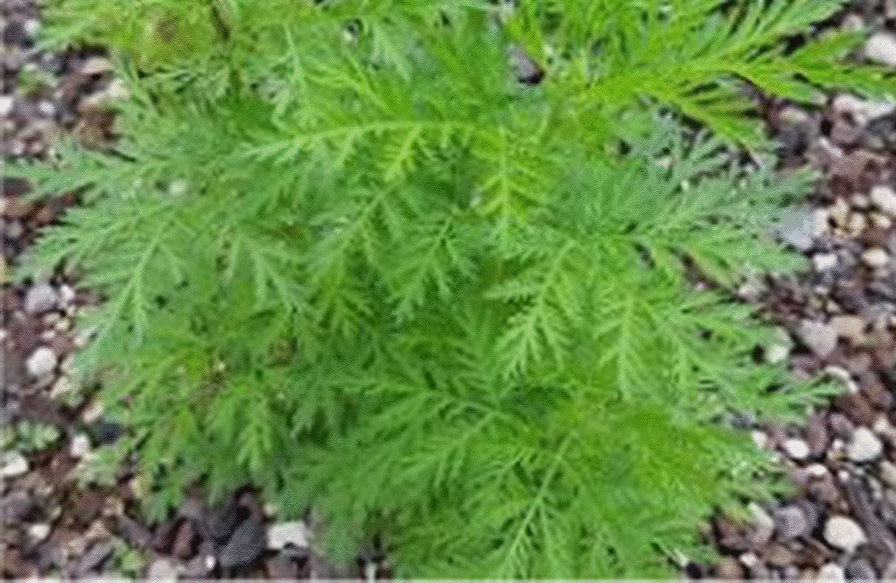
*A. annua*

*Artemisia annua* (Fig. 1) is known as sweet wormwood, sweet annie or annual wormwood in English. Botanically, it has been classified as Family—Asteraceae, Genus—*Artemisia*, Species: *annua* [30, 31].

As shown in Fig. 1, the plant is a large vigorous weedy annual shrub often reaching more than 2 m tall, usually ribbed single-stemmed with alternate branches and stem covered with fine, silky grey-green hairs [46]. It naturally grows to 30–100 cm high but cultivated plants may reach 200 cm high, and is widely distributed in the temperate, cool temperate and subtropical zones (mainly in Asia) of the world [47]. It originated from China and grows mainly in the middle, eastern and southern parts of Europe and in the northern, middle and eastern parts of Asia. However, a few countries are currently cultivating *A. annua* on both large and small scale, such as China, Kenya, the United Republic of Tanzania, and other countries in Africa (including Uganda), and altitudes ranging 1000–1500 m are favourable for its growth [48].

**Fig. 2:**
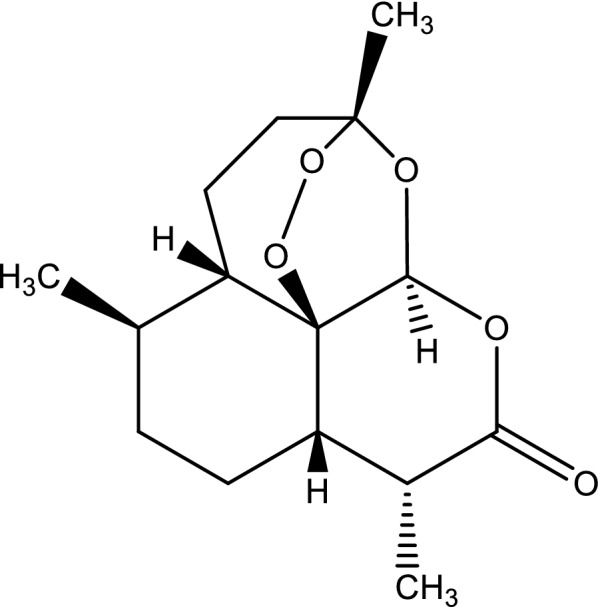
Chemical structure of artemisinin

*Artemisia annua* (called Qinghao—Chinese) has long been used in China as a herbal remedy with its first documentation dating 168 BC [48]. Its first record for malaria was made by Ge Hong in 341 AD. Li Shizen also wrote in his Pharmacopoeia in 1596 that qinghao cures cold and hot fevers. Based on this, the Chinese scientists led by Zhenxing Wei and You-Tou Li developed extraction methods to finally isolate its active compound, artemisinin (Fig. 2) in 1972 [45].

Since the isolation of the active ingredient, artemisinin has influenced the current treatment of malaria. In most African countries, ACT was introduced right on time when the parasite had already developed resistance against the previous drugs, including chloroquine, quinine and others. The trends over the years on this novel plant is fascinatingly filled with disagreements among various researchers on some issues and agreements on other issues, especially between researchers from herbal regimen and those from synthetic regimen. The anti-malarial literature on the plant ranges from in vitro, in vivo in animals to several human clinical trials.

The earliest clinical study conducted on *A. annua* leaf infusion was performed 20 years ago as a pilot trial in a rural primary health care scheme, involving a district hospital and three health centres, in the eastern Democratic Republic of the Congo (DRC) from February to December 2001 [49]. In the study, extract groups (2) received 5 g (A5) and 9 g (A9) of the leaf powder prepared in 1 L per day and the control group were on quinine and later changed to chloroquine. A 77% cure rate was reported in A5 group and 70% in A9 while the positive control led to 91% by day 7. Parasite recrudescence was observed in the infusion groups after day 7 though there was no clear method used to distinguish recrudescence from new infections. However, there was a rapid improvement of malaria symptoms in artemisia groups, leading to a conclusion that, “malaria monotherapy with tea preparation cannot be recommended for treatment because of recrudescence as well as concerns of possible artemisinin resistance. A similar study was done in Tanzania in 2002—2003 with same doses of *A. annua* infusion but sulfadoxine/pyrimethamine used as control [50]. Compared to the previous trial [49], the number of participants available by day 7 was lower (4–8, compared to 39–43). However, the cure rate reported in this work (70% & 77.7% for 5 g and 9 g doses, respectively) was similar to the study from DRC. Another similarity with both studies is that the plant materials were all collected from Germany and a high rate of recrudescence.

Contrary to the findings in the two clinical studies above, a cure rate of 91.8% was reported in an Ethiopian survey that assessed the use of the plant for malaria treatment among locals with no major adverse effects [51]. The researchers recommended that a policy and regulatory mechanisms to integrate herbal medicines to modern health care system should be established and adopted. But these findings were only based on experiences of people, with no scientific methods to prove the cure and may not be applicable for informing policy. Interestingly, these findings were corroborated by a pharmacovigilance study in Kenya and Uganda which revealed that, over 3000 cases of presumed malaria (250 children and 54 pregnant women in 1st trimester) were treated using *A. annua* [52]. However, the latter study reported poor compliance by children to the infusion because of bitterness and vomiting. Two miscarriages were reported among the pregnant patients. In a randomized Ugandan clinical trial [53], consumption of the *A. annua* tea infusion (5 g) once a week significantly reduced the risk of suffering more than one episode of malaria in 9 months by 55%. No serious side effects were reported except the bitter taste associated with the plant. Another study [54] supported the efficacy reported of *A. annua* tea infusion for malaria treatment. The researchers reported a potent activity demonstrated by the infusion in vitro against both chloroquine resistant (W_2_) and sensitive (D10) strains (IC50 (ug/ml): 1.11 ± 0.21 for D10 and 0.88 ± 0.35 for W_2_). With the minimal concentration of artemisinin in the infusion (0.18 ± 0.02% of the leaf powder), which was too low to be responsible for the observed activity, they postulated the possibility of artemisinin acting synergistically with other ingredients in the extract to give such an impressive effect.

Despite the efficacy of the crude plant extracts reported above, the WHO in 2012 ruled out the use of *A. annua* plant material in any form including capsules and tea for malaria treatment or prevention [55]. The international regulatory body decision was based exclusively on two trials described previously [35, 36], stating that the reported clinical outcomes were unsatisfactory with malaria recrudescence, low dose of the infusions compared to the recommended ACT dose may promote anti-malarial resistance, and reported interactions between artemisinin and other compounds in the infusions are unsatisfactory. The WHO recommended that an extensive fundamental and clinical research be done to demonstrate that the non-pharmaceutical dosage forms of the plant are safe and effective for malaria, and that their use would not lead to development of artemisinin-resistant parasite. A follow-up study on the possibility of synergism among various compounds in the plant was conducted [56], different varieties of the plant were tested in comparison with pure artemisinin. It was observed that the IC_50_ of all the *A. annua* tea infusions were not significantly different from that of pure artemisinin, and thus in agreement with the WHO analysis. The researchers, therefore, concluded that, “artemisinin seems to be the only active anti-malarial agent in *A. annua*, but they did not comprehensively explain the similarity in the IC_50_’s between the extract and the pure drug since the extract obviously has lower quantity of the active and would be expected to show lower activity if their conclusion is to be justified.

In a comprehensive literature review on the use of *A. annua* for malaria management [37], reviewers highlighted discrepancies in the studies used for informing the above WHO decision. They also presented several studies that provided evidence of efficacy of the plant infusion for malaria especially those that proved biopharmaceutical interaction viz., a number of compounds from flavonoids, terpenes polysaccharides and others that improved absorption of artemisinin and some exhibiting anti-plasmodial activities. They suggested that the tea could play a critical role in malaria prophylaxis to reduce incidence of malaria in different communities or in temporary relief of malaria as the patient buys time to access a hospital or a clinic stocked with ACT. More scientific evidences have also emerged recently all supporting the synergism between artemisinin and other compounds. For example, in a combination of artemisinin with three other components in high contents in the *A. annua* (arteannuin B, arteannuic acid, and scopoletin), a sharper reduction in parasitaemia (93%) compared to the pure artemisinin (30%) in animal model was reported [17]. This indicated clear synergism among the different compounds. The pharmacokinetic studies showed increased absorption in the combination groups. The findings imply that specific components in the plant might offer a possibility to develop new artemisinin-based natural combination therapy for malaria treatment. In a similar study, arteannuin B was reported to inhibit biotransformation of artemisinin through inhibition of CYP3A [57]. This observed effect of arteannuin B explains the enhanced anti-plasmodial potency of the plant extract, but the synergism did not reduce the rate of recrudescence. Therefore, there is still the need to combine the extract with other anti-malarial agents to prolong activity and alleviate recrudescence.

The limitation of *A. annua* tea infusion in malaria treatment by parasite recrudescence was further evidenced in another randomized controlled trial, which used artemether-lumefantrine as control. The study reported negative parasitaemia in the tea infusion within few days but recrudescence surfaced on day 14 and 28. The researchers also attributed the observed activity to interaction between artemisinin and other components (possibly flavonoids), and recommended combination of the extract with other agents to extend therapeutic action [31]. In contrast to a large-scale randomized, double blind and controlled study showing 91% and 100% cure rates in children and adults, respectively, compared to 50 and 30% (children and adults respectively) for the standard control (artesunate-amodiaquine – ASAQ) [58], no recrudescence was reported after 28 days in the extract group. Due to strong criticism of the study findings [35], the publication was retracted. Despite continued challenges in acceptability of *A. annua* extract for malaria prophylaxis or treatment, more evidence on its effectiveness continues to surface. Another study in 2020 [59] reported that, the malaria prophylaxis provided by the plant infusion that was thought to last for few weeks actually extends for months and years. This was explained by an observation that the IgE induced *Artemisia* consumption remained for months on the skin. Another researcher described therapeutics based on isolated molecules such as quinine or its derivative chloroquine, artemisinin or its derivatives (artesunate), as having higher tendencies to cause drug resistance than crude plant extracts having complex chemical composition [60]. Therefore, standardization of crude extract formulations will not only lead to introduction of phytopharmaceuticals into the conventional health care mainstream, but also prevent any possible drug resistance which is common with synthetic drugs.

#### Vernonia amygdalina

**Fig. 3:**
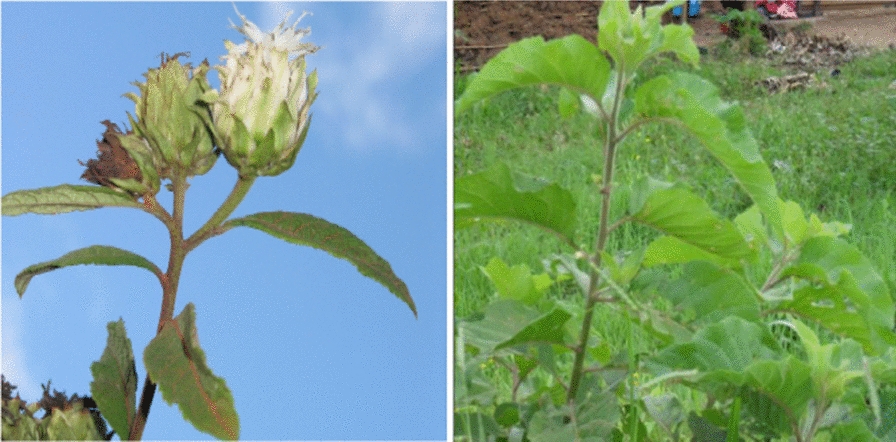
*V. amygdalina* [62]

*Vernonia amygdalina* (Fig. 3) is a popular African vegetable that grows as a shrub or small tree indigenous to Central and East African including Uganda [61]. Ecologically and botanically, the plant has been previously described as follows [51]. It grows up to 10 m tall along rivers and lakes, in forests margins, woodland and grassland up to 2800 m altitude, in regions where mean annual rainfall is 750- 2000 mm The bark is light grey or brown; fissured, brittle branches. Leaves lanceolate oblong; up to 28 × 0 cm, but usually 10–15 × 4–5 cm. Flower heads thistle like, small, creamy white, 10 mm long, grouped in dense heads, axillary and terminal, forming large flat clusters, 15 cm in diameter, sweetly scented.

The plant is commonly referred to as bitter leaf but in Uganda, it has various local names; Luganda—Mululuuza, Lunyankole—Mubirizi, Acholi—Labori, Lugishu—Mululisi, Madi—Okelo-okelo, Iteso—etutum, and Lugbara—Echero [63]. It is used for treatment of multiple diseases in Uganda including schistosomiasis, amoebic dysentery, and gastrointestinal problems. Masaba reported reported the chewing of the pith of the plant by Chimpanzees for treating parasitic infections and determined the in vitro anti-plasmodial activity of aqueous and acetone–water extract obtaining IC_50_ of 76.7 µg/ml and 25.5 µg/ml respectively [64]. Another in vitro activity was tested in fresh isolated *P. falciparum* parasites from patients in Nigeria [65]. The researchers reported IC_50_ of 11.2 µg/ml for ethanolic leaf extract and 13.6 µg/ml for aqueous extract. These anti-plasmodial activities were higher than the study by Masaba [64]. The difference could be attributed to geographical locations (Nigeria vs Uganda) and parasite strains (isolates from Nigeria vs school child chloroquine sensitive strain—Uganda).

Several in vivo animal anti-malarial studies have also been conducted to validate the above in vitro findings (Table 4). The variations in the activities from different studies may emanate from many factors including geographical locations, route of administration, different extraction methods, and antimalarial method (prophylactic, curative and suppressive). The highest activity reported so far with the standard Peter’s four-day suppressive test was from the *V. amygdalina* collected from Uganda, that is, 75.15% at 400 mg/kg [66]. A previous study in Uganda [61], gave a similar activity at an even lower dose but the extract was administered by IP (intraperitoneal) route.

**Table 4:** In vivo anti-malarial activities of *V. amygdalina* leaf

Efficacy (% suppression)	Plant collection site	Extract	Dose (mg/kg)	Route	Parasite strain	References
73.9	Uganda	Aq.	200	i.p	*Plasmodium berghei*	[61]
32.47, 35.40, and 37.67%	Ethiopia	80% methanolic extract	200, 400 and 600 respectively	oral	*P. berghei* (ANKA strain)	[141]
68.8, 69.3, 70.3% (suppression test, curative and prophylactic resp.)	Ethiopia	Aq.	600	Oral	*P. berghei*	[69]
69.2, 70.8, and 71.5% (suppression test, curative and prophylactic resp.)	Ethiopia	Hydro-methanolic	600	Oral	*P. berghei*	[69]
67.0%	Botwana	Ethanolic	500	NS	P. berghei	[142]
63.92% and 75.13%	Uganda	Aq.	200 and 400 respectively	Oral	Plasmodium berghei, Strain ANKA, MRA-311	[66]

In addition to the in vivo animal studies validating the anti-malarial efficacy of the plant, two clinical studies have also been conducted in Uganda [56, 57]. The first was done in Bushenyi at Rukararwe Partnership Workshop for Rural Development (RPWRD), and the product was coded AM—which refers to antimalaria *V. amygdalina* leaf powder [67]. Complete parasite clearance was achieved only in one case, but the geometric mean of parasite count declined significantly by day 7 (5540/µl day 0 to 511/ µl day 7). There was also marked symptomatic improvement in 17/19 patients. No severe side effects were observed, and the most common minor ones reported included vomiting, abdominal pain, nausea, and bitter taste. These side effects affected the compliance of patients leading to six (6) dropouts. However, the study recommended a larger randomized controlled trial to determine whether the symptomatic improvement was the result of AM treatment or of natural immunity to malaria. The second trial [32] conducted in Kasese district (Uganda) reported adequate clinical response (ACR) on day 14 in 67% of the cases. However, complete parasite clearance occurred in only 32% of those with ACR, and of these, parasite recrudescence occurred in 71%. Just like the previous study, no severe side effects were reported except nocturia, insomnia and cough. According to the researchers, *V. amygdalina* is moderately effective and further work is necessary to establish the optimum dosage regimen, possibly in combination with other anti-malarial agents. A follow up study was conducted to validate this claim [68], and it reported 80.71% chemosuppression from a combination of *V. amygdalina* (125 mg/kg) and chloroquine (5 mg/kg), in mice against chloroquine resistant clones of *Plasmodium berghei* strain ANKA. The study concluded that, *V. amygdalina* leaf extract dose – dependently restored the efficacy of CQ against CQ resistant *P. berghei* malaria in mice. In another study in Uganda, a combination of *V. amygdalina* with *A. annua* achieved 100% parasite clearance in mice model [39]. There was still a challenge of shorter survival time (10.67 ± 1.09 days) compared to more than 30.0 ± 0.0 days for the ACT, P = 0.000. The study concluded that, “the *V. amygdalina* – *A. annua* petroleum ether extract combination shows promise for use as an herbal artemisinin combination against malaria, however the survival times need improvement to match that of the ACT”.

The phytochemicals responsible for above-described activities of *V. amygdalina* have been identified, isolated and their anti-plasmodial effect determined. They include (with their IC_50_) sesquiterpene lactones: vernodalin (4.0 µg/ml), vernolide (8.4 µg/ml), vernodalol (4.2 µg/ml), and hydroxyvernolide (11.4 µg/ml); and also steroid glycoside: vernonioside B1 (46.1 µg/ml) [38, 70]. The bitter taste of the plant leaf decoction is attributed to the steroid glycosides (vernoniosides A_1_ – A_4_ and B_1_ – B_4_). The LD_50_ of the leaf decoction has been reported to be > 2000 mg/kg [61, 62].

#### Microglossa pyrifolia

**Fig. 4:**
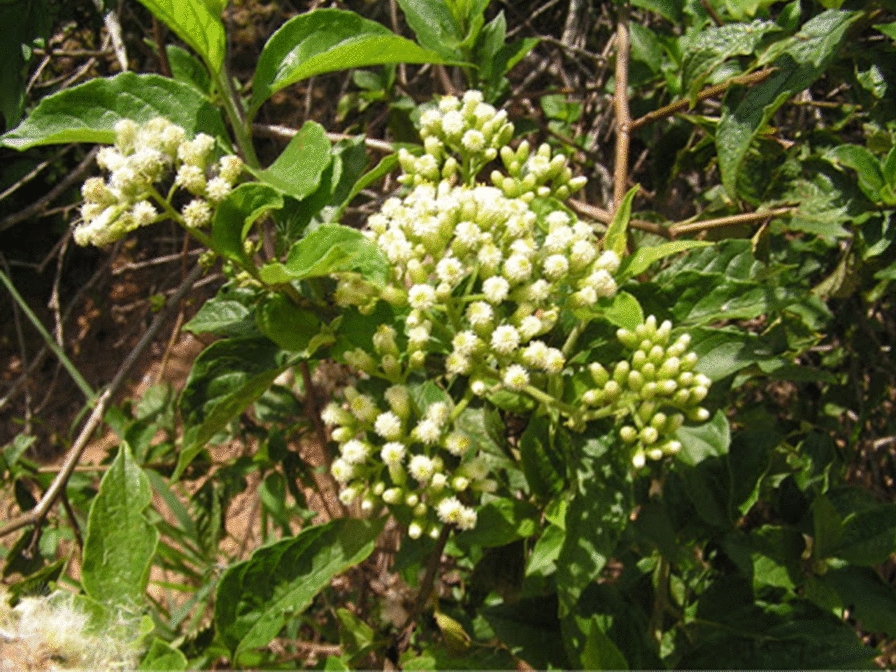
*M. pyrifolia* [78]

*Microglossa pyrifolia* (Fig. 4) is an erect or scandent shrub that grows up to 5 m high, occurring throughout tropical Africa and Asia [71]. The plant is locally known as Kafugakande in Uganda – Luganda [72], Nyabungodide in Kenya [73]. Diola in Senegal and Gambia, and Bulom in Sierra Leon [71]. The leaves are simple alternate, carried by a short petiole (10–15 mm long). The leaf blade is oval, 5 to 10 cm long and 2.5 to 4 cm wide.

The plant leaf is used locally in Uganda for treatment of malaria and several in vitro anti-plasmodial studies have provided evidence supporting this use (Table 5). The plant leaves have demonstrated very potent (IC_50_ < 2 µg/ml) anti-plasmodial activity according to the RITAM ranking of in vitro efficacy across different *Plasmodium* strains and countries. Interestingly in Uganda, the aqueous leaf extract also showed a very potent activity [72]. A similar finding was reported earlier in a Kenyan study, though the extract used in the latter was methanol [73]. The promising anti-plasmodial activity of this plant has been attributed to presence of several terpenoids, such as E-phytol, 1,3- hydroxyoctadeca-9Z, 11E, 15-trien-oic-acid and 6E-geranylgeraniol-19-oic-acid, which exhibited IC_50_ values between 2.5 and 13.7 µg/ml [74]. The higher IC_50_ values shown by these active compounds compared to the crude extracts is pointing to possible combined effects (synergism or additive) among the compounds in the extract against the malaria parasite. In a recent mini-review on the anti-plasmodial activities of various plants around the world, *M. pyrifolia* leaf extracts (ethyl acetate and aqueous solvents) ranked among the best three in terms of their lower IC_50_ indicating higher potency [75]. The researchers attributed the potency of the plant to 6E-geranylgeraniol-19-oic-acid, tannins and other polar compounds. The 6E-geranylgeraniol-19-oic-acid is also known to be present in aqueous extracts despite its lipophilic character [74].

**Table 5:** Anti-plasmodial activity of *M. pyrifolia*

Efficacy (IC_50_—µg/ml)	Plant collection site	Extract	Parasite strain	References
0.05 ± 1.24	Buyiga-Buwama, Mpigi district at PROMETRA (Uganda)	Ethyl acetate	NF54	[72]
0.03 ± 1.72			FCR_3_	
0.05 ± 1.24		Aqueous	NF54	
0.23 ± 1.78			FCR_3_	
1.59 ± 0.07	L. Victoria (Kenya)	Methanol	D6	[65, 73, 143]
2.50 ± 0.15			W-2	
10.5	Ghana	PE – EtoAc (1:1, w/v)	Pow	[74]
13.1			Dd_2_	
33.1 ± 4.1	Ivory Coast	Ethanol	FcB1	[144]
4.2 ± 1.9	Rwanda	Methanol	3D7	[76]
1.5 ± 0.1		CH_2_Cl_2_		
14.3 ± 2.1		Aqueous		

In terms of safety, the plant leaf methanolic extract showed a higher cytotoxicity antiplamodial ratio (CAR) of 1578.0 (chloroquine sensitive strain, D-6) and 946.8 (chloroquine resistant strain, W-2), which are indicative of weak toxicities [73]. In a similar study, 89.7% reduction of ATP levels have been reported with the methanolic extract of the plant indicating a hepatotoxic effect [76]. This finding also agreed with the study [43], that listed the plant among those associated with liver fibrosis in Rakai—Uganda. The study mentioned that plants of *Microglossa* family contain diterpenoids known to cause liver toxicity. In addition, herbs in Asteraceae family contain pyrrolizidine alkaloids which are associated with veno-occlusive liver disease [77]. Unfortunately, there are no in vivo toxicity studies on the plant to determine its LD_50_ for guiding dose selection for efficacy studies.

Most of the anti-malarial studies on the plant stopped at in vitro despite its extremely potent anti-plasmodial activity. Therefore, there is a need to conduct in vivo anti-malarial activities for this plant since its anti-plasmodial potency has already been demonstrated in different countries and strains of the malaria parasite. It is also possible that the plant (in combination) may successfully improve anti-malarial efficacy of *A. annua* and/or *V. amygdalina*, especially the challenge of parasite recrudescence. Addition of the plant extract in small doses in such a combination may also control its potential liver toxicity.

## Conclusion

There are many plants used in Uganda for management of malaria and ~ 33% have been either studied for efficacy and safety locally or outside the country with the plant materials collected from within the country. Most of these efficacy studies cover in vitro anti-plasmodial and in vivo chemosuppressive tests, but some plants such as *M. pyrifolia* that have demonstrated very potent anti-plasmodial effects have not been studied in vivo for both efficacy and safety. *Artemisia annua*, *V. amygdalina*, *C. longa*, *A. africana*, and *A. afra* are among the few plants broadly studied for malarial treatment up to clinical trials. However, the clinical data on *C. longa* stops at bioavailability and the study on *A. afra* was retracted due to alleged inaccuracies in the study. *Aspilia africana* possesses good clinical outcomes but no active compounds have been reportedly detected or isolated for easy standardization of its extracts if considered for product formulation.

This leaves *A. annua* and *V. amydalina* with known active anti-malarial compounds as the main plants that can be considered now for product developments utilizing the crude extracts. They are already being cultivated, the leaves can be harvested sustainably and extracted by aqueous solvent making it cost effective for large scale product manufacturing. But their clinical outcomes are limited by malaria parasite recrudescence. It is envisaged that addition of potent aq. extracts of *M. pyrifolia* to a combination of the two plants, *A. annua* and *V. amydalina* in development of polyherbal anti-malarial formulations may result in complete clearance of the parasite from the body. However, this needs to be explored and extensively studied.

## Data Availability

Nil.
